# Supplementary material for: Clinical and biological correlates of morning serum cortisol in children and adolescents with overweight and obesity
Source: PLoS One. 2021 Oct 20;16(10):e0258653. doi: 10.1371/journal.pone.0258653 (PMC8528324; doi:10.1371/journal.pone.0258653)
Supplement: Attachment — Submitted filename: Response to Reviewers.docx [file pone.0258653.s002.docx]

**Response to the reviewers’ comments**

We thank the reviewers for their valuable comments and constructive suggestions. Please find our point-by-point answers enclosed.

**Reviewer #1**

Summary and overall impression:

The study is a descriptive retrospective analysis studying fasting hyperglycemia, dyslipidemia, hyperfibrinogenemia as primary parameters/ or outcomes in overweight/obese children with high cortisol compared with those with normal cortisol level. However, more secondary parameters / or outcomes were mentioned later in the method, results, and discussion and were not referred to in the study aim and introduction.

Major changes:

1. Aim: To add an additional aim outlining the secondary cardio-metabolic parameters /outcomes that were assessed in the study.

The different parameters that were studied were specified in the revised version.

“*Our aim was to clinically characterize overweight or obese youth with high morning cortisol and to study whether these children and adolescents are at higher risk for metabolic syndrome conditions such as high fasting glucose or triglycerides, insulin resistance, low HDL cholesterol, or high blood pressure compared to overweight or obese youth with normal cortisol levels. “*

2. Methods and patients: It will be better if you define the cutoff levels of cardio-metabolic parameters in the method and demonstrate the normal and high range. E.g., define hyperglycemia or dyslipidemia and so on. Also, there was not a cut-off level differentiating obese from overweight for further analysis.

We thank the reviewer for this suggestion. A supplementary paragraph “calculations and definitions” was added in the revised version.

*“Body mass index (BMI) was calculated by dividing the weight in kilograms by height in meters squared (kg/m²). Anthropometric values were expressed as standard deviation scores (SDS) or z-scores [z-score = (value - mean)/SDS] for gender and age according to national reference data (19). Blood pressure values were expressed as SDS for gender, age, and height according to international reference data (20). HOMA-IR was calculated according to the formula: fasting insulin (pmol/L) x (fasting glucose (mg/dL) / 2430. LDL cholesterol was calculated using the formula: total cholesterol - HDL cholesterol - (triglycerides / 5).*

*Overweight was defined by a BMI z-score > 1.3 and obesity by a BMI z-score > 2. An elevated fasting glucose concentration was defined by a fasting glucose above 100 mg/dl. Dyslipidemia was defined by a HDL cholesterol < 35 mg/dl, triglycerides > 150 mg/dl or a LDL cholesterol > 135 mg/dl. Elevated blood pressure was defined by either a systolic or diastolic blood pressure SDS > 2. Insulin resistance was defined by a HOMA-IR > 4. An elevated morning serum cortisol concentration was defined by a value above 180 µg/L and a decreased concentration by a value below 62 µg/L, while an elevated fibrinogen was defined by a value > 400 mg/dl.”*

3. Sample Size: How the sample size was tested and assured that it was large enough to generate reliable results?

In the statistical analysis the calculation of the sample size has been included in the revised manuscript. “*Using a two sided test, 5% significance level test (α=0.05) with power 80% power (β=0.2), the required sample size is approximate 198 to detect Spearman Rank order of 0.2 between cortisol and glucose.* “

Statistical Analysis:

The flow of the statistical analysis was towards proving the objective of the study, which is the correlation between elevated cortisol and hyperglycemia, dyslipidemia, hyperfibrinogenemia and hyperleptinemia.

In the introduction and in the discussion of the revised manuscript, we have clearly stated our aims to characterize both clinically and biologically obese children and adolescents with elevated morning serum cortisol. We therefore added a supplementary table with the clinical characteristics (table 3).

Table 1: presented the anthropometric characteristics and compare them between boys and girls. The characteristics were stated in terms of the median and the range. The listed characteristics are usually normally distributed, and I suggest using mean ± sd to their measurements and parametric test for the comparisons.

We thank the reviewer for this suggestion. We now present these data as means ± SD.

Table 3 and table 5 presented the main outcomes of the study. In table 3 the biological characteristics were compared over the three subgroups of the population: low cortisol (<62 μg/L), normal cortisol (62-180 μg/L) and elevated cortisol (>180 μg/L). Through this classification it was recognized that the elevated cortisol subgroup represents about 20% of the total sample. The Kruskal Wallis p-values were not stated for significant comparisons between the three subgroups. The analysis was based on pairwise comparisons that identified the significance difference between the elevated cortisol subgroup from the lower cortisol subgroup with respect to fasting glucose, fibrinogen and leptin. The authors though did not mention the test on which the pairwise comparisons were made in their analysis on page 7.

Kruskal Wallis p-values were added in the revised version. The paired comparisons were done by the Mann Whitney U test, as mentioned now in the paragraph “statistical analysis “.

The author has to take these points on board and rearrange the measured risk factors into primary and secondary in the aim and introduction sections, also rearrange results and tables display accordingly. Discussion has to go in systemically in the same flow of results display.

We have clearly stated in the introduction and in the discussion of the revised manuscript our aims to characterize both clinically and biologically obese children and adolescents with elevated morning serum cortisol. We therefore added a supplementary table with the clinical characteristics (table 3). In the results and discussion first the clinical and subsequently the biological associations are described.

**Reviewer #2**

I am pleased to write my review on "Correlates of morning serum cortisol in children and adolescents with overweight and obesity" where authors investigated whether hypercortisolemia conferred an increased risk for fasting hyperglycemia, dyslipidemia, hyperfibrinogenemia, and hyperleptinemia in overweight children and adolescents. The article is well written and easy to understand but I would like to ask few concerns related to this study.

1. The size of population is not huge and why did the authors selected this particular time period for this study?

In the revised version, we added a sample size calculation.

“*Using a two sided test, 5% significance level test (α=0.05) with power 80% power (β=0.2), the required sample size is approximate 198 to detect Spearman Rank order of 0.2 between cortisol and glucose.* “

This particular time period was chosen due to methodical reasons. In this period, no changes occurred in the analysis technique of the body composition and of the blood sample analyses.

2. What do the authors think why morning cortisolemia was found to correlate positively with fasting glucose, irrespective of fasting insulin?

The higher fasting glucose in subjects with hypercortisolism can be explained by a stimulated gluconeogenesis / impaired glucose effectiveness (Khani S, Tayek JA. Cortisol increases gluconeogenesis in humans: its role in the metabolic syndrome. Clin Sci (Lond). 2001;101(6):739-47.). We added this in our discussion.

“*The association between serum cortisol and glucose persisted after correction for serum insulin, confirming that cortisol excess impairs glucose intolerance, by decreasing not only the insulin action, but also glucose effectiveness (Nielsen MF, Caumo A, Chandramouli V, Schumann WC, Cobelli C, Landau BR, et al. Impaired basal glucose effectiveness but unaltered fasting glucose release and gluconeogenesis during short-term hypercortisolemia in healthy subjects. Am J Physiol Endocrinol Metab. 2004;286(1):E102-10).”*

3. Have the authors check role of depression or anxiety in these participants for elevated morning cortisol concentrations?

No psychological analysis of the subjects was performed in this retrospective study.

4. Did the authors study role of sleep duration as it could be a reason for elevated morning cortisol level?

Our data on sleep duration were incomplete, thus not allowing an analysis. We added this point in the discussion. *“ The duration of the obesity, the presence of obstructive sleep apnea syndrome or sleep deprivation, and the emotional status of the included subjects could not be studied due to incomplete data. “*

5. As children were in Pediatric Obesity Clinic for obesity management, can author comment on usage of any drugs or obesity management techniques which can result in stress?

No other investigations were performed before collection of the blood samples. Also, none of the subjects took any medication.

**Reviewer #3**

The study is relevant and has scientific importance. Data were well analyzed. The results are similar to other published studies, which gives this manuscript importance. It is interesting that the authors continue to look for new answers to the initial questions.

We thank the reviewer for his/her appreciation of our study.

**Reviewer #4**

General impression:

The study was based on retrospective medical record of children and adolescents with overweight or obesity problems seeking for obesity management. It was conducted to assess the relationship between hypercortisolemia and the risk of hyperglycemia, dyslipidemia, hyperfibrinogenemia, and hyperleptinemia in overweight children and adolescents. However, the MM, results and discussion cover diverse parameters, which were not specified precisely in the research objectives. The conclusion was not aligned with the main objective of the research.

Reviewing the manuscript revealed that the research idea has a potential for publication. However, setting objectives, MM, data analysis and presentation, depth of the discussion and engagement with published literatures and the conclusion all need rigorous amendments to achieve an acceptable level for publication. The current level of manuscript written language and flow of ideas hinders its suitability for publication. The extensive grammatical mistakes affected manuscript presentation in general causing it to be inappropriate for publication at this level.

Confidential detailed comments for the editors/authors:

Title: Needs modifications

I would suggest modifying the title to best reflect the research objective(s) and the measured parameters.

We thank the reviewer for this suggestion and changed the title to “Clinical and biological correlates of morning serum cortisol in children and adolescents with overweight and obesity”.

Abstract: Needs modifications

Along with the changes recommended in the various sections of the manuscript, you may notice that the abstract will demand subsequent amendments.

The abstract was revised to show more clearly the clinical as well as the biological aims and results.

Introduction: Needs modifications

The introduction is very brief and does not cover all the measured substantial parameters. More emphasis should be done on reviewing published literature pertaining the relationship between hypercortisolemia and increased risk of hyperglycemia, dyslipidemia, hyperfibrinogenemia, and hyperleptinemia and the other measured parameters. You may like to elaborate on the effect of overweight/obesity on hypercortisolemia and how it affects the stated cardiometabolic parameters. Engagement with published literatures can highlight what was/were the outcomes of the published research and hence you can emphasize the originality of your research. The aim of the research must be supported with statements indicating the rationale behind selecting the studied parameters to assess the link between morning serum cortisol and hyperglycemia, dyslipidemia, hyperfibrinogenemia, and hyperleptinemia in overweight and obese patients. You may like to think of adding other objective(s) that can be related to the parameters analyzed in the study but not precisely stated in the objectives or focused on in the introduction.

We have changed the introduction by providing background information on the mechanisms relating hypercorticism with insulin resistance, fasting glucose, arterial hypertension and abdominal fat accumulation.

*“These increased serum cortisol levels have been related to overactivation of the hypothalamic-pituitary-adrenal (HPA) axis (12). However, decreased serum cortisol levels secondary to an increased cortisol metabolic rate have also been reported (13, 14). While the evidence for systemic hypercortisolism in obesity is not conclusive, it is well established that present chronic cortisol excess causes multiple physiological changes: increased insulin resistance (by interfering with insulin signaling (15)), higher fasting glucose levels (by decreasing insulin action and glucose effectiveness (16)), higher blood pressure readings (by activating the mineralocorticoid receptor and increasing the vascular responsiveness (17)), and more abdominal fat accumulation (by promoting adipocyte differentiation through glucocorticoid receptor signaling, which are more abundant in visceral compared to subcutaneous fat (18)).”*

We also added the shortcomings of the previous studies investigating the relationship with the components of the metabolic syndrome in the introduction.

*“Previous work reporting on serum morning cortisol was either restricted to Latino youth under 14 years of age (6), obese infants (7), or excluded subjects with arterial hypertension (8). Most of the studies lacked data on body composition (4-9).”*

Material and Methods (Methods and patients): Needs modifications

The flow of this section is logic as it started with anthropometry and body composition analysis to obtain an overview of the studied sample. This was followed by blood sample collection and analysis, and finally the statistical analysis. However, this section needs to be revised. The following are some examples that need to be taken care of:

• Sample size: adequate sample size (n) should be calculated and specified precisely before proceeding with data analysis.

We have added a sample size calculation in the revised version.

*“Using a two sided test, 5% significance level test (α=0.05) with power 80% power (β=0.2), the required sample size is approximate 198 to detect Spearman Rank order of 0.2 between cortisol and glucose. “*

• The blood sample collection and analysis sections lacks rudimentary information that makes the work difficult to replicate and the results to be reproducible. E.g “Glucose, fibrinogen, total cholesterol, LDL-cholesterol, HDL- cholesterol, and triglycerides were measured by automated methods (Cobas 8000 C702)” it is not clear how samples were prepared for the automated analyzer and the analysis was performed. “Hormone levels were measured using commercial immunoassays”, it is not clear, which hormone they measured and what is the type and the manufacturer of the commercial immunoassays. More emphasis should be exerted on presenting details about the method of the analysis carried out. If details are not specified, then methodology should be supported with literatures.

Thank you for pointing out the absence of sufficient detail in this section. We adapted our text to include methodological information to improve the reproducibility of our results:

*“Glucose was measured in lithium-heparin (LiHep) plasma using the hexokinase assay on a Cobas 8000 C702 / Roche Diagnostics platform. Total cholesterol, LDL-cholesterol, and HDL-cholesterol were measured in LiHep plasma using the cholesterol oxidase/peroxidase assay (Cobas 8000 C702). Triglycerides were measured in LiHep plasma using the glycerol kinase/glycerol phosphate oxidase/peroxidase assay (Cobas 8000 C702). Fibrinogen was measured in sodium-citate plasma using the turbidimetric clotdetection assay on an ACL Top Family / Instrumentation Laboratory platform. Insulin was measured by electrochemiluminescence immunoassay (Cobas 8000 e801), leptin by ELISA (Wallace Victor2 TRF counter, Perkin Elmer), and cortisol by electrochemiluminescence immunoassay (Cobas 8000 e801).”*

• The collected data were subjected to Kruskal Wallace test, the Mann-Whitney U test, and the Chi square test to compare differences between groups. However, this is not always reflected in the results sections. E.g. Table 3 and the accompanied narrative.

We added the p-values of the Kruskal Wallace test to the corresponding tables.

• Clinical and biological characteristics indicates that the sample used in the study may be non-homogeneous, e.g. wide age groups, wide BMI and body fat % range. Did you perform homogeneity test between groups before performing the above analysis?

We used non-parametric tests to account for non-Gaussian distribution of several parameters.

• What about the differences between overweight and obese patients? Children and adolescents? Can we consider them as one group?

Only 35 (15 %) patients were severe obese, defined by a BMI z-score > 3. Serum cortisol did not correlate with age, pubertal stage or BMI z-score, allowing the correlation analysis in the whole group.

Results: Needs modifications

In this part of the manuscript, you have presented several parameters and performed data analysis to achieve the main objective. Here are few comments that you may like to think about:

• The research depends on retrospective medical record of a wide age groups, which includes children and adolescents with diverse physiological status. Don’t you think, it would be more logic to divide the sample based on age groups with distinct physiological status?

We do agree that a wide age range was studied. But, no effect of age on serum cortisol was found. Listing all results separately for the different age groups would result in too many tables to be incorporated in the manuscript.

• In the characterization of overweight children and adolescents with an elevated morning serum cortisol section and Table 3: the p-values need to be indicated to reveal the significant or non-significant differences between groups. I believe more emphasis should be attained on the difference between normal and elevated/reduced cortisol to meet the objective(s) of the study.

We thank the reviewer for this suggestion. The p-values have been added to table 3 and 4.

According to Table 3: are the results presented in this section related to obese patients only? Did you exclude overweight patients? Can you precisely identify the difference(s) between overweight and obese patients?

Overweight as well as obese patients were include in the analysis. Overweight was defined as BMI SDS > 1.3 and obesity by a SDS > 2 (see methods). There were no significant differences in serum cortisol between obese and overweight subjects (see results). Therefore, we chose to incorporate the available data of both obese and overweight patients in this table.

• In the correlates of serum cortisol with clinical characteristics and auxological measurements section: important data which reveals the differences between different pubertal stages are not shown, any reason for that? What is/are objective(s) of measuring the correlation between serum cortisol and clinical characteristics and auxological parameters? How this will help in achieving your objective.

The great majority of our subjects were prepubertal, leaving only 10 patients in Tanner stage 4. Further subgroup analysis of the Tanner stages are not indicated as subgroups are not equally distributed and certain subgroups are underpowered.

Incorporating all results separately for age groups and pubertal stages would result in too many tables to include in the manuscript. More important, age neither pubertal stage were correlated with serum cortisol in our studied population.

In our revised introduction, we now clearly state our two objectives to characterize the obese children and adolescents, both clinically and biologically: Our aim was to clinically characterize overweight or obese youth with high morning cortisol and to study whether these are at higher risk for metabolic syndrome conditions such as high fasting glucose or triglycerides, insulin resistance, low HDL cholesterol, or high blood pressure compared to overweight or obese youth with normal cortisol levels.

• In the section which revels the correlates of serum cortisol with cardiometabolic parameters, what does the “r” value indicates? Do you think that there are strong relationships between the measured parameters? Refer to the conclusion.

*The r values are the Rs values in the Spearman rank correlation test. We replaced the “r” by “Rs”.*

• I think presenting data in the tables as means ± SD, is more meaningful compared to the median.

We adapted this and now present our data as means ± SD in table 1 and 2.

Discussion: Needs modifications

The discussion lacks deep and critical analysis of the research findings. You may like to provide strong and deep justification(s) to support your arguments in relation to the findings. Engagement with published literatures should be used to support your argument, it should not be presented as literature search to present general information.

We discussed the prevalence of elevated cortisolemia and the association with fasting glucose in detail as these were the most important findings of our study. As we did not find any significant correlation with clinical characteristics, this aspect of our study could is not discussed in detail. We further elaborated on the remaining positive correlation between glucose and cortisol after controlling for insulin.

Conclusion: Needs modifications

The conclusion is not satisfactory. It does not highlight the main outcome(s) of the research. You may like to link it with the objectives and the measured parameters to draw a clear and precise conclusion. In addition, can the results presented in this study support your conclusion? i.e. “these increased levels correlated with higher fasting glucose concentrations” while r = 0.193?

We do think the conclusion gives an answer to the aims of our study, which are also now stated in the adapted title. Also, we added the “weak” association between the morning glucose and cortisol in the revised version.

*“In conclusion, elevated morning serum cortisol levels were present in 20 % of overweight or obese children and adolescents. Obese children and adolescents with high serum cortisol concentrations could not be differentiated on clinical or biological grounds from those with normal serum cortisol. Morning serum cortisol concentrations were found to correlate only weakly with fasting glucose concentrations, irrespective of insulin resistance. Further study exploring long-term cardiometabolic consequences of hypercortisolemia in childhood obesity is needed.*

References: Needs more engagement with relevant literatures

You need to follow the referencing style recommended by the journal both in-text and in the list of references. Unified style is must.

Extra relevant literature on mechanisms relating hypercortisolism and metabolic syndrome conditions was provided in the revised manuscript. We now use Vancouver style referencing (with brackets) throughout the manuscript.
